# Vascular architecture characters and risk factors analysis of unstable moyamoya disease

**DOI:** 10.3389/fneur.2024.1398007

**Published:** 2024-05-31

**Authors:** Liming Zhao, Ruiyu Wu, Bingqian Xue, Tao Gao, Yang Liu, Yuxue Sun, Gaochao Guo, Tianxiao Li, Chaoyue Li

**Affiliations:** ^1^Department of Neurosurgery, Zhengzhou University People’s Hospital, Henan Provincial People’s Hospital, Zhengzhou, China; ^2^Department of Neurosurgery, Henan University People’s Hospital, Henan Provincial People’s Hospital, Zhengzhou, China

**Keywords:** moyamoya disease, cerebral revascularization surgery, unstable moyamoya disease, intracranial and extracranial vascular bypass, perioperative management

## Abstract

**Background:**

In some MMD patients, the digital subtraction angiography (DSA) examination found, occlusion in the ipsilateral internal carotid artery or middle cerebral artery, accompanied by the formation of numerous moyamoya vessels. Conversely, the contralateral internal carotid artery or middle cerebral artery shows signs of stenosis without the presence of moyamoya vessels. Notably, cerebral perfusion studies reveal a similar or even more severe reduction in perfusion on the occluded side compared to the stenotic side. Importantly, clinical symptoms in these patients are typically attributed to ischemia caused by the stenotic side. This condition is referred to as unstable moyamoya disease (uMMD).

**Objective:**

This clinical research focuses on evaluating risk factors related to MMD and developing strategies to minimize postoperative complications. The study aims to analyze vascular characteristics and identify potential risk factors in patients with uMMD.

**Methods:**

The authors reviewed consecutive cases with complete clinical and radiological documentation of patients who underwent surgery between January 2018 and June 2023. Univariate analysis and multivariate logistic regression analysis were employed to understand the risk factors and prognosis of postoperative complications in uMMD.

**Results:**

Postoperative complications were retrospectively analyzed in 1481 patients (aged 14 to 65). Among them, 1,429 patients were assigned to the conventional treatment group, while 52 were in the unstable moyamoya disease group. The uMMD treatment group showed a significantly higher incidence of early postoperative complications such as RIND, cerebral infarction, and cerebral hemorrhage (*p* < 0.05). Univariate and multivariate logistic regression analyses were conducted on the postoperative complications of 52 uMMD patients. Initial symptoms of stenosis ≤50% (univariate: *p* = 0.008, multivariate: *p* = 0.015; OR [95% CI] =23.149 [1.853–289.217]) and choosing occluded side surgery (univariate: *p* = 0.043, multivariate: *p* = 0.018; OR [95% CI] =0.059 [0.006–0.617]) were identified as significant risk factors for postoperative neurological complications.

**Conclusion:**

Compared to the conventional treatment group, uMMD has higher complication rates, with vascular stenosis degree and surgical side selection identified as significant risk factors. A comprehensive understanding of preoperative clinical symptoms and vascular characteristics in moyamoya disease patients, coupled with the formulation of rational surgical plans, contributes positively to decreasing postoperative mortality and disability rates in uMMD.

## Introduction

Moyamoya disease (MMD) is a chronic progressive steno-occlusive cerebrovascular disease, affecting the terminal internal carotid artery (ICA), and the proximal segments of the middle cerebral artery (MCA) and anterior cerebral artery (ACA) together with the development of new collateral vessels (1). However, these pathological anastomoses are imperfect, leading to the well-known clinical manifestations of the disease such as ischemic strokes or transient ischemic attacks (TIAs), and the possibility of intracerebral hemorrhage due to the fragility of these new vessels (2, 3).

Traditionally, MMD refers to a bilateral vascular affection, in some MMD patients, the digital subtraction angiography (DSA) examination found, occlusion in the ipsilateral ICA or MCA, accompanied by the formation of numerous moyamoya vessels (4, 5). Conversely, the contralateral ICA or MCA shows signs of stenosis without the presence of moyamoya vessels. Notably, cerebral perfusion studies reveal a similar or even more severe reduction in perfusion on the occluded side compared to the stenotic side. Importantly, clinical symptoms in these patients are typically attributed to ischemia caused by the stenotic side. This condition is referred to as unstable moyamoya disease (uMMD) (6). These characteristics likely reflect the complex relationship of many factors influencing the cerebral blood flow (CBF) and perfusion (7–12).

Due to this florid clinical and radiological disparity, and the presence of different angiographic patterns, treatment of uMMD patients is challenging. Since, they commonly suffer strokes while waiting for surgery or during the perioperative period. In this study, we retrospectively analyzed the clinical data, imaging features, treatment strategies, and prognosis of patients with uMMD. The clinical features and risk factors were summarized to provide guidance for the diagnosis, treatment, and management of these patients.

## Methods

### Study cohort

From January 2018 to June 2023, we retrospectively collected clinical and radiological data of 2,376 patients with MMD according to the Chinese Expert Consensus on Diagnosis and treatment of MMD (13) admitted at the Moyamoya Disease Research and Treatment Center of the Henan Provincial People’s Hospital in Zhengzhou – China.

We identified 1,481 patients that met the following inclusion criteria: (a) age within 14–65 years old, (b) clinical and angiographic diagnosis of MMD, (c) Complete perioperative clinical and radiological data. (d) Surgical treatment through direct superficial temporal artery to middle cerebral artery (STA-MCA) bypass + encephalo-duro-myo-synangiosis (EDMS).

The 52 patients uMMD included should meet the criteria (a, b, c, d) and also satisfy the following conditions: (e) Digital subtraction angiography demonstrating occlusion of one ICA, ACA or MCA, and progressive stenosis of the contralateral ICA, ACA or MCA, without the presence of moyamoya vessels, (f) The clinical symptoms of the patient were mainly on the side of stenosis rather than on the side of occlusion.

Patients that failed to preclude the giving inclusion criteria and that presented with severe cognitive dysfunction after cerebral hemorrhage or cerebral infarction, as well as patients with cardiopulmonary disease, hyperthyroidism, systemic lupus erythematosus, antiphospholipid antibody syndrome, peripheral arteritis, Sjogren’s syndrome were excluded from the study cohort. Additionally, patients with symptoms related to the occluded ICA were excluded as well.

### Radiological evaluation

The relevant preoperative and postoperative radiographic images (computed tomographic angiography, digital subtraction angiography, magnetic resonance enhanced perfusion imaging or CT perfusion imaging) were retrieved from the hospital’s digital archiving system.

**Figure 1:**
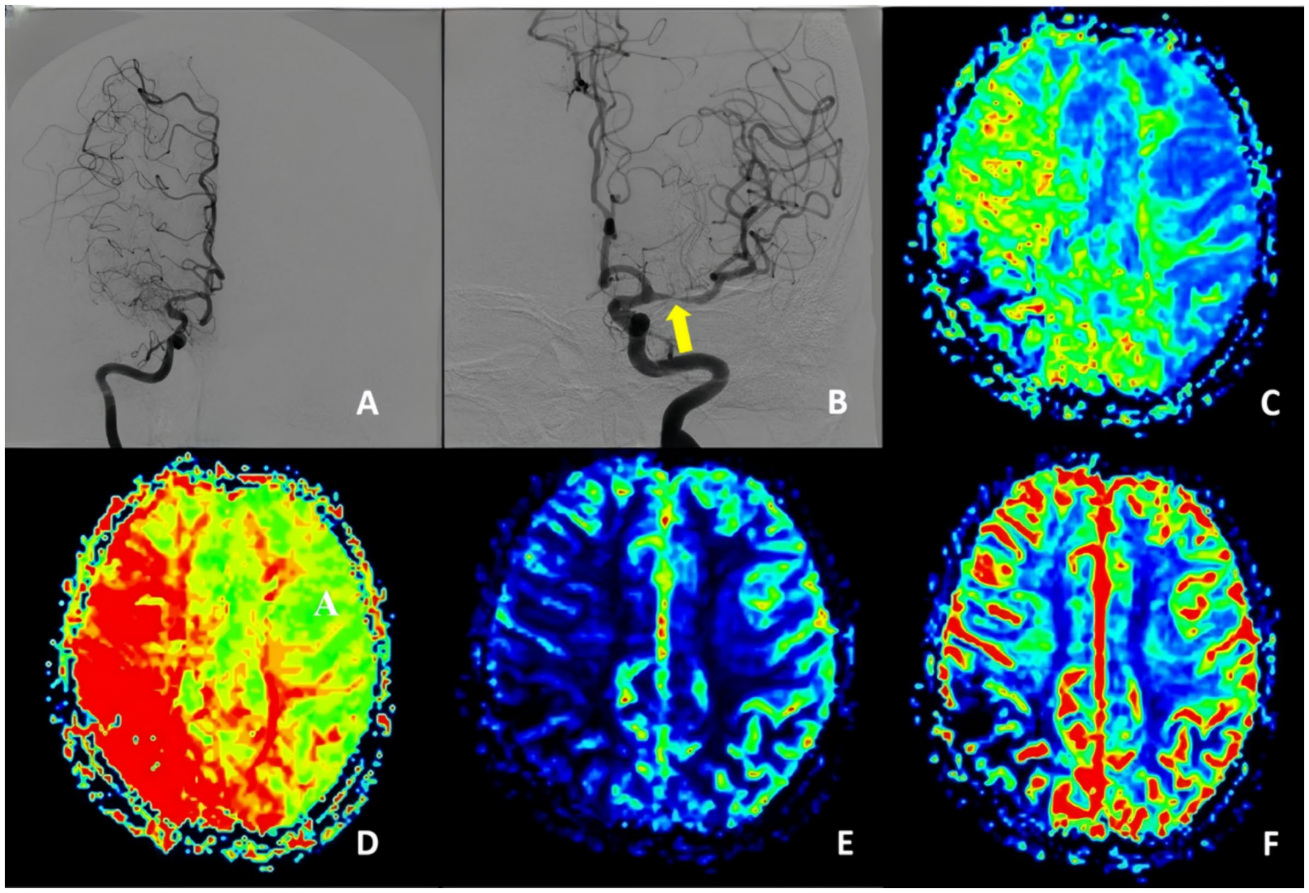
Unstable moyamoya disease. **(A)** Middle cerebral artery occluded and presence of moyamoya vessels. **(B)** Middle cerebral artery stenosis. **(C–F)** PWI show MTT, TTP, CBF, and CBV indicated bilateral ischemia, with the degree of ischemia being less pronounced on the left stenotic side compared to the right occluded side. PWI, perfusion-weighted imaging; CBV, cerebral blood volume; CBF, cerebral blood flow; DSA, digital subtraction angiography; MTT, mean transit time.

We defined uMMD as the presence in DSA of ipsilateral ICA or MCA occlusion together with the development of moyamoya vessels and the presence of progressive stenosis on the contralateral ICA or MCA without the evidence of moyamoya vessels, cerebral perfusion studies reveal a similar or even more severe reduction in perfusion on the occluded side compared to the stenotic side (Figure 1) clinical symptoms in these patients are typically attributed to ischemia caused by the stenotic side.

### Surgical procedure

The revascularization side was selected according to clinical symptoms (presence of transient ischemic attacks or ischemic strokes) and radiological affection.

All patients underwent a combined revascularization procedure which included indirect flow augmentation by EDMS and direct bypass through the STA-MCA bypass. Prior performing the anastomosis, all patients underwent indocyanine green (ICG) video angiography and FLOW800 analysis to ensure placing the anastomosis in a low perfusion area in correlation with the radiological findings. We decided to perform STA-MCA bypass according to the hypoperfusion extension. Perioperatively, patient’s blood pressure and PaCO_2_ (between 35 and 40 mmHg) were strictly controlled to decrease the risk of unexpected complications (10, 14).

### Immediate outcome and complication rate

Postoperative outcome was assessed by a neurosurgeon at discharge according to the modified Rankin scale (mRS) (15). We classified the outcome according to the mRS as good (0–2), or poor (3–6). We reviewed the immediate morbidity, including possible surgical and neurological complications, as well as the localization (ipsilateral or contralateral side) of postoperative strokes.

### Ethics and statistics

This study has the approval of the Henan Provincial People’s Hospital Ethics Committee (HIRB-2019-329 Chinese moyamoya disease registry). Due to the retrospective nature of this manuscript written informed consent was waived. Statistical analysis was conducted using SPSS software (SPSS26.0 IBM. US). Continuous variables were compared using a *t*-test, while categorical variables were assessed using the appropriate method: Pearson chi-square test, Fisher’s exact test, or Mann–Whitney test *p-*value <0.05 was considered statistically significant. To account for multiple variables, multivariate logistic regression analysis was performed on factors achieving *p* < 0.05 in the univariate analysis, as well as factors deemed clinically relevant based on experience.

## Results

### Patients’ characteristics

**Figure 2:**
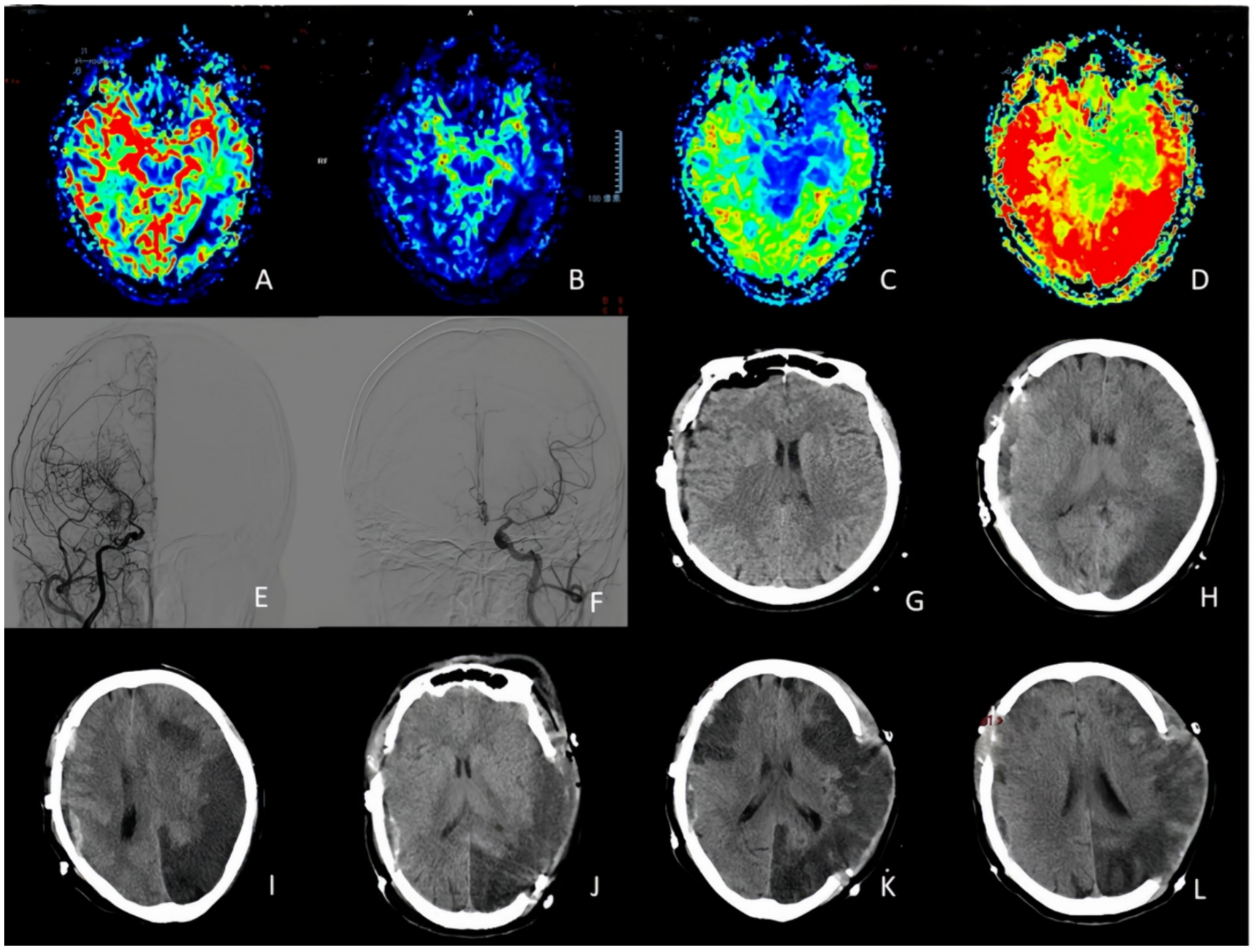
A 63-years-old woman who experienced right side lower extremity weakness one prior surgical treatment. **(A,B)** PWI indicated CBV decreased in the left temporal occipital lobe and right frontal and parietal lobe, and CBF decreased in the right cerebral hemisphere and left temporal and occipital lobe. **(C,D)** PWI showed MTT and TTP delayed in right cerebral hemisphere and left temporal occipito-parietal lobe. **(E,F)** DSA suggested occlusion of the right internal carotid artery with moyamoya vessels. Left middle cerebral artery and anterior cerebral artery stenosis. **(G–I)** CT changes on postoperative day, postoperative day 1, and postoperative day 3 indicated a severe cerebral infarction on the non-operative side (stenotic side). **(J–L)** On the third day, the patient underwent left side decompressive craniectomy, and CT findings were obtained at postoperative day 1, 3 and 10.

From January 2018 to June 2023, we retrospectively collected clinical and radiological data of 2,376 patients with MMD according to the Chinese Expert Consensus on Diagnosis and treatment of MMD (13) admitted at the Moyamoya Disease Research and Treatment Center of the Henan Provincial People’s Hospital in Zhengzhou – China. According to the enrollment criteria, 1,481 patients aged between 14 and 61 years were included in this study, and their clinical characteristics are summarized in Table 1. The proportion of female patients was slightly higher than that of male patients (50.3% vs. 49.7%). There were 266 cases (18.0%) of hemorrhagic stroke, 1,215 cases (82.0%) of ischemic stroke, with a higher incidence on the right side compared to the left side (51.9% vs. 48.1%) (Figure 2).

**Table 1:** Baseline characteristics of the included patients.

	Value
No. of patients	1,481
Age, (years)^a^	51(42,57)
Sex^b^	
Male	736(49.7)
Female	745(50.3)
Premorbid history^b^	
Hypertension	548(37.0)
Diabetes	311(20.2)
Smoker	636(42.9)
Drinker	459(31.0)
Onset type^b^	
TIA	578(39.0)
Hemorrhagic	266(18.0)
Infarction	637(43.0)
mRS at admission^b^	
0–2	985(66.5)
3–5	496(33.5)
Surgical side^b^	
Right	769(51.9)
Left	712(48.1)
Suzuki stage^b,c^	
I	0(0.0)
II	281(19.0)
III	589(39.8)
IV	392(26.5)
V	219(14.8)
VI	0(0.0)

^a^Median (Interquartile Range). ^b^Percentage (%). ^c^In this and successive tables, Suzuki stage refers to the Suzuki stage of the surgically treated side.

### Analysis of postoperative complications

**Figure 3:**
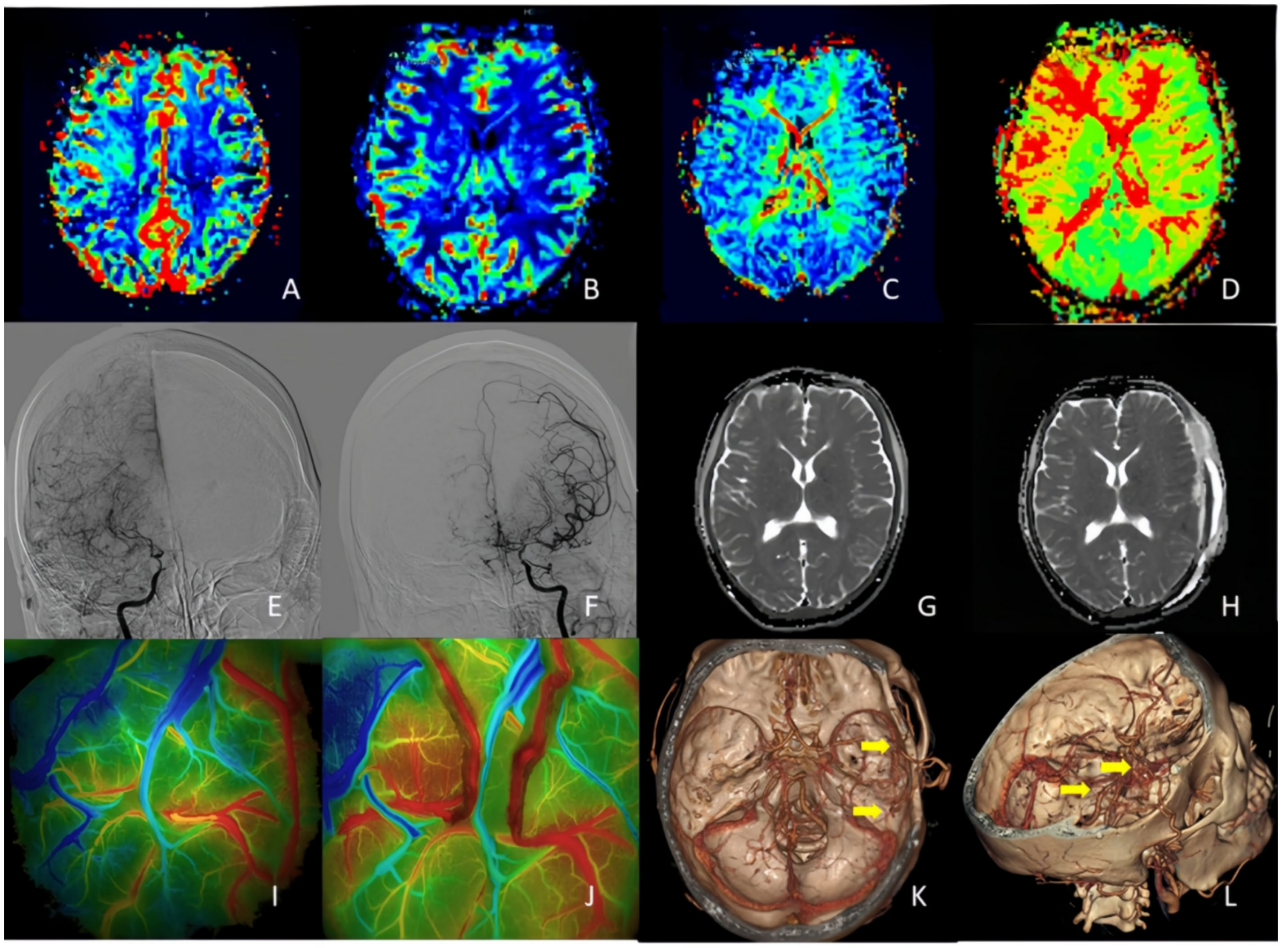
A 32-year-old woman who presented with right side hemiparesis that gradually worsened. Postoperatively, on day 4 the patient developed transient motor aphasia speech impairment which improved at 10 day. **(A,B)** PWI indicated that bilateral CBV and CBF were symmetrical, and no significant increase or decrease was observed. **(C,D)** PMI showed MTT and TTP delayed in bilateral occipital lobe and right frontotemporal lobe. **(E,F)** DSA indicated right anterior cerebral artery and right middle cerebral artery occlusion with moyamoya vessels and severe stenosis of the left middle and anterior cerebral arteries. **(G,H)** On postoperative day 4, follow-up MRI performed due to transient motor aphasia showed no significant difference as compare to preoperative images. **(I,J)** FLOW800 angiography results before and after double–branch STA-MCA bypass. **(K,L)** 3D-CTA reconstruction showed the location of double-branch bypass. ADC, apparent diffusion coefficient; 3D-CTA, three-dimensional CT angiography.

According to the grouping criteria of 1,481 patients with moyamoya disease, 1,429 patients were assigned to the conventional treatment group, while 52 patients were assigned to the unstable moyamoya disease group. No statistically significant differences were found between the two groups in gender, age, preoperative clinical symptoms, MRS Score, and Suzuki stage (all *p* > 0.05), indicating that the baseline data were essentially consistent and comparable (Figure 3).

One thousand four hundred and twenty nine patients were assigned to the conventional treatment group, while 52 patients were assigned to the unstable moyamoya disease group. The treatment group for uMMD exhibited a significantly higher incidence of early postoperative complications such as RIND, cerebral infarction, and cerebral hemorrhage (*p* < 0.05) (Table 2).

**Table 2:** Analysis of clinical data and postoperative complications in patients with moyamoya disease.

	All patients	MMD	uMMD	*p*-value
	(*n* = 1,481)	(*n* = 1,429)	(*n* = 52)	
Age, years^a^	51(42,57)	50(42,57)	49.90 ± 10.88	0.578
Sex^b^				
Male	736(49.7)	710(49.7)	26(50.0)	0.964
Female	745(50.3)	719(50.3)	26(50.0)	
mRS at admission				
0–2	985(66.5)	946(66.2)	39(75.0)	0.191
3–5	496(33.5)	483(33.8)	13(25.0)	
Personal history^b^				
Diabetes	311(21.0)	305(21.3)	6(11.5)	0.088
Hypertension	548(37.0)	524(36.7)	24(46.2)	0.164
Smoking	636(42.9)	616(43.1)	20(38.5)	0.506
Drinking alcohol	459(31.0)	445(31.1)	14(26.9)	0.518
Onset type^b^				0.205
TIA	578(39.0)	554(38.8)	24(46.2)	
Infarction	637(43.0)	613(42.9)	24(46.2)	
Hemorrhagic	266(18.0)	262(18.3)	4(7.6)	
Suzuki stage^b,c^				0.429
I	0(0.0)	0(0.0)	0(0.0)	
II	281(19.0)	274(19.2)	7(13.5)	
III	589(39.8)	565(39.5)	24(46.2)	
IV	392(26.5)	376(26.3)	16(30.8)	
V	219(14.8)	214(15.0)	5(9.6)	
VI	0(0.0)	0(0.0)	0(0.0)	
Operative side^b^				0.778
Left	712(48.1)	688(48.1)	24(46.2)	
Right	769(51.9)	741(51.9)	28(53.8)	
Postoperative complications				<0.001^*^
RIND	89(6.0)	87(6.1)	2(3.8)	
Infarction	35(2.4)	17(1.2)	18(34.6)	
Hemorrhagic	17(1.1)	15(1.0)	2(3.8)	
Epileptic	25(1.7)	24(1.7)	1(1.9)	
mRS at postoperative				0.035
Improve	410(27.7)	400(28.0)	10(19.2)	
Unchanged	1,010(68.2)	974(68.2)	36(69.2)	
Deteriorate	61(4.1)	55(3.8)	6(11.5)	

^a^Median (Interquartile Range). ^b^Percentage (%). ^c^In this and successive tables, Suzuki stage refers to the Suzuki stage of the surgically treated side. ^*^P < 0.05.

### Risk factor analysis of uMMD

Further analysis of early postoperative complications in the 52 patients with uMMD included 23 cases with neurological complications and 29 cases without neurological complications. There were no statistically significant differences in mRS scores, gender, age, blood sugar and blood pressure levels, smoking and drinking history, Suzuki stage, and clinical symptoms between the two groups (*P* > 0.05), indicating comparability.

Through univariate analysis and multivariate logistic regression analysis: the Initial symptoms of stenosis ≤50% (univariate: *p* = 0.008, multivariate: *p* = 0.015; OR [95% CI] = 23.149 [1.853–289.217]; Table 2) and choosing occluded side surgery (univariate: *p* = 0.043, multivariate: *p* = 0.018; OR [95% CI] =0.059 [0.006–0.617]) were identified as significant risk factors for postoperative neurological complications.

### Surgical results, complications and outcome

Preoperative DSA imaging was utilized to assess the degree of stenosis in the affected blood vessels. Out of the 15 cases with stenosis ≤50%, 11 cases (73.3%) experienced postoperative complications, involving 4 cases affecting solely the stenotic side, 2 cases affecting solely the occluded side, and 2 cases affecting both sides. Additionally, two patients experienced intracerebral hemorrhage, and one developed postoperative epileptic seizure. Among the 37 patients with vascular stenosis >50%, 12 patients (32.4%) encountered postoperative complications, with 4 cases affecting solely the stenotic side, 4 cases affecting solely the occluded side, and 2 cases affecting both sides. Additionally, 2 patients experienced RIND. No wound-healing issues or infections were noted within this cohort (Table 3).

**Table 3:** Analysis of risk factors for postoperative complications in uMMD.

	Postoperative complications		*P*-value		OR (95% CI)
	Present (*n* = 23)	Absent (*n* = 29)	Univariate	Multivariate	
Age, years^a^	47.30 ± 11.46	51.97 ± 10.13	0.126		
Sex^b^			1.000		
Male	12(52.2)	14(48.3)			
Female	11(47.8)	15(51.7)			
mRS at admission			0.524		
0–2	16(69.6)	23(79.3)			
3–5	7(30.4)	6(20.7)			
Personal history^b^					
Diabetes	2(8.3)	4(12.9)	0.682		
Hypertension	9(37.5)	15(48.4)	0.412		
Smoking	8(34.8)	12(41.4)	0.776		
Drinking alcohol	5(21.7)	9(31.0)	0.539		
Onset type^b^			0.479		
TIA	13(56.5)	11(37.9)			
Infarction	9(39.1)	15(51.7)			
Hemorrhagic	1(4.3)	3(10.3)			
Suzuki stage^b,c^			0.898		
I	0(0.00)	0(0.00)			
II	3(13.0)	4(13.8)			
III	11(47.8)	13(44.8)			
IV	7(30.4)	9(31.0)			
V	2(8.7)	3(10.3)			
VI	0(0.00)	0(0.00)			
Vascular stenosis^b^			0.008^*^	0.015^*^	23.149(1.853–289.217)
≤50%	11(47.8)	4(13.8)			
>50%	12(52.2)	25(86.2)			
Operative side^b^			0.043^*^	0.018^*^	0.059 (0.006–0.617)
Stenotic	4(17.4)	13(44.8)			
Occlusion	19(82.6)	16(55.2)			

^a^Mean ± SD. ^b^Percentage (%). ^c^In this and successive tables, Suzuki stage refers to the Suzuki stage of the surgically treated side. ^*^P < 0.05.

According to the preoperative cerebral perfusion examination results, 35 patients underwent surgery on the occluded side based on the degree of ischemia, resulting in 19 patients (54.3%) experiencing postoperative complications. Among these, 17 patients developed cerebral infarction (7 cases affecting solely the stenotic side, 6 cases affecting solely the occluded side, and 4 cases affecting both sides). Furthermore, 2 patients suffered from intracerebral hemorrhage. Seventeen patients chose to undergo surgery on the side with stenosis based on preoperative clinical symptoms, with 4 patients (23.5%) experiencing postoperative complications. One patient developed cerebral infarction on the stenotic side, while 2 patients experienced RIND, and 1 patient developed postoperative epileptic seizure. No wound-healing issues or infections were observed within this group.

## Discussion

The MMD is a chronic progressive steno-occlusive cerebrovascular disease affecting the terminal portion of the ICAs, and the proximal segments of the ACA and MCA, characterized by the formation of collateral vessels in order to compensate for reduced cerebral blood flow and perfusion (1, 2, 8, 11). Frequently, these neovascular attempts often fail causing ischemic strokes, or due to its fragility may cause intracerebral hemorrhage.

Revascularization procedures are widely accepted in the treatment of MMD patients because they may decrease the rate of ischemic events and improve cerebral blood flow and perfusion (10, 16, 17), effectively reducing the incidence of stroke. Untreated MMD patients have a higher recurrence rate of cerebrovascular events and poorer prognosis when compared to those patients undergoing surgical treatment, regardless of the disease type manifested at the initial attack (18–20). Independently of the asymptomatic or symptomatic clinical presentation, and the definitive/probable diagnosis, MMD patients demonstrated progression of the disease in up to 20% of cases in the non-surgically treated hemisphere, and ischemic strokes, TIAs or intracranial hemorrhages likely occur in about half of the cases (21) (29 Japanese guidelines 263 page). Thus, suggesting a more aggressive management of those patients. However, possible perioperative complications such as TIA, RIND, cerebral infarction, cerebral hemorrhage, and epilepsy can significantly impact patient prognosis. Therefore, a strict preoperative evaluation of patients and the development of appropriate surgical plans can truly enhance neurological function in patients safely and effectively.

Patients with MMD progress slowly in the early stages, exhibiting relatively stable hemodynamics. However, long-term ischemia significantly reduces the reserve capacity of cerebral blood flow, functional reserve, and metabolic reserve. In severe cases, local blood flow conflict may occur, resulting in “local high perfusion and peripheral low perfusion,” thus increasing the risk of stroke events (15, 17). However, patients typically present clinical symptoms attributable to ischemia on the stenotic side, termed uMMD. In this retrospective analysis of 1,481 MMD patients, postoperative complications were significantly higher in those with uMMD, reaching 44.23% (23/52), compared to 10.01% (143/1429) in the conventional group, and this difference was statistically significant. This is primarily due to the unstable condition of intracranial blood vessels on the stenotic side, progressive symptom deterioration, and the compromised brain function and metabolic reserve capacity of these patients. Additionally, some patients may experience the stolen blood syndrome, exacerbating ischemia in the narrow lateral brain tissue and potentially leading to severe stroke events (18). Therefore, precise preoperative assessment and management, prudent selection of surgical protocols, and early postoperative complication prevention are crucial for patients with uMMD.

In patients with MMD on one side of blood vessel occlusion and contralateral vascular stenosis, although cerebral perfusion indicates severe ischemia on the occlusive side, the clinical symptoms are mainly caused by ischemic attack on the narrow side, including migraine, dizziness, limb numbness and weakness, slurred speech, blurred vision, etc. Imaging on the stenotic side indicates that, although ischemia is not severe, there is no compensatory blood vessel in the brain or no effective compensatory blood vessel has been formed yet, making it susceptible to low blood pressure, blood volume, and vasospasm threshold, resulting in TIA, RIND, or cerebral infarction. Clinical manifestations in such patients are more instructive than imaging findings, and the incorrect selection of the surgical side accelerates the probability of this event, which is also a key factor in our decision on the surgical side. Frequent TIA symptoms before surgery suggest that the stenotic side is experiencing extensive ischemia of the entire cerebral cortex and unstable blood flow, posing a high risk of stroke events before effective and stable self-compensation is established.

The number of patients who fully meet the criteria for uMMD in clinical practice is relatively small. Consequently, this study could not conduct large-scale prospective cohort studies, potentially introducing bias into the results. However, enhancing the focused management of uMMD, formulating reasonable diagnosis and treatment plans, and surgical strategies for various risk factors are crucial for preventing postoperative complications and reducing the disability and mortality rates of MMD surgery.

## Conclusion

Moyamoya disease is a chronic steno-occlusive cerebrovascular pathology that often shows clinical and angiographic disparity. Rapid progression of the disease (repeated TIAs or cerebral infarction within 1 month before surgery) as well as absence of moyamoya vessels suggest the presence of hemodynamic instability. Surgical treatment and management should be directed to the symptomatic and stenotic hemisphere of patients with bilateral compromise (one side vascular occlusion and one side vascular stenosis), since this decrease the risk of postoperative cerebral infarction and neurological deficits.

## Data availability statement

The original contributions presented in the study are included in the article/supplementary material, further inquiries can be directed to the corresponding authors.

## Ethics statement

The studies involving humans were approved by the Ethics Committee of Henan Provincial People’s Hospital. The studies were conducted in accordance with the local legislation and institutional requirements. Written informed consent for participation was not required from the participants or the participants’ legal guardians/next of kin in accordance with the national legislation and institutional requirements.

## Author contributions

LZ: Conceptualization, Project administration, Writing – original draft. RW: Data curation, Investigation, Writing – original draft. BX: Data curation, Investigation, Writing – original draft. TG: Conceptualization, Writing – review & editing. YL: Writing – original draft. YS: Conceptualization, Writing – original draft. GG: Conceptualization, Writing – original draft. TL: Writing – review & editing. CL: Funding acquisition, Writing – review & editing.
